# Clinical Implications of Mounjaro (Tirzepatide) for Breast Cancer Detection and Management: A Narrative Review

**DOI:** 10.7759/cureus.111445

**Published:** 2026-06-24

**Authors:** Raafat Mady, Habib Tafazal, Haitham Soliman, Ayman Ramadan, Mohamed Hajaj

**Affiliations:** 1 Department of Breast Surgery, Kettering General Hospital, Kettering, GBR; 2 Department of Breast Surgery, Blackrock Clinic, Dublin, IRL; 3 Department of Medical Oncology, Kettering General Hospital, Kettering, GBR; 4 Radiology Department, Breast Cancer Center, American Hospital, Dubai, ARE

**Keywords:** breast cancer detection, mounjaro, obesity associated mammary tumor, tirzepatide glp-1, weight-loss

## Abstract

Tirzepatide, marketed as Mounjaro, is a dual glucose-dependent insulinotropic polypeptide (GIP) and glucagon-like peptide-1 (GLP-1) receptor agonist that is widely used to treat type 2 diabetes and obesity. As more women presenting to screening and symptomatic breast clinics receive tirzepatide, questions have emerged regarding how rapid pharmacologic weight loss may influence breast lump detection, mammographic density, and breast cancer risk. This narrative review summarises tirzepatide pharmacology, its effects on adiposity, expected changes in breast composition with weight loss, oncologic data on breast cancer risk and outcomes, and radiological implications for mammography, ultrasound, and MRI. Randomised-trial data and meta-analyses currently show no clear evidence of increased breast cancer incidence with tirzepatide or GLP-1 receptor agonists. Preclinical studies in obesity-associated breast cancer models have demonstrated reduced mammary tumour progression following tirzepatide-induced weight loss and metabolic improvement; however, the clinical relevance of these findings remains uncertain. In the clinic, GLP-1 receptor agonist use in women with breast cancer has been associated with clinically meaningful weight loss without short-term safety signals. Weight loss decreases breast volume and subcutaneous fat, often making pre-existing benign lesions more palpable and altering mammographic density and parenchymal patterns, but available data do not indicate reduced imaging accuracy. Clinicians should maintain standard triple assessment, recognise that new palpable nodularity may reflect unmasked benign tissue, and reassure patients that current clinical evidence has not demonstrated an increased breast cancer risk associated with tirzepatide use. High-quality prospective imaging and oncology studies are needed to define long-term effects.

## Introduction and background

Glucagon-like peptide-1 (GLP-1) receptor agonists and dual-incretin therapies have transformed the management of hyperglycaemia and obesity, with tirzepatide producing substantial mean weight reductions in phase 3 clinical trials [1-5]. As tirzepatide is increasingly prescribed to women within breast screening age groups and to breast cancer survivors experiencing treatment-related weight gain [6-10], breast clinicians are increasingly encountering patients whose breast tissue composition and physical examination findings may evolve rapidly during pharmacologically induced weight loss. Obesity is a well-established risk factor for postmenopausal breast cancer and is associated with more advanced stage at diagnosis, poorer oncologic outcomes, and technical challenges in breast imaging and clinical examination [9,11-14].

Traditional breast cancer risk models, mammographic density assessment systems, and imaging workflows have largely been developed in populations with relatively stable body composition [14]. Rapid pharmacologic weight loss therefore introduces uncertainty regarding the interpretation of new palpable findings, interval density changes, and evolving imaging characteristics in women receiving tirzepatide. This narrative review synthesises current evidence at the intersection of metabolic endocrinology, breast oncology, radiology, and breast surgery, with particular focus on the implications of tirzepatide-associated weight loss for breast tissue composition, imaging interpretation, clinical assessment, and oncologic practice.

## Review

Search strategy and study selection 

This narrative review was conducted to evaluate the potential effects of tirzepatide and other GLP-1 receptor agonists on breast tissue composition, breast mass detection, breast cancer risk, breast imaging, clinical assessment, surgical decision-making, and oncologic management. As incretin-based therapies are increasingly prescribed for obesity and type 2 diabetes in women across a broad age range, understanding their implications for breast practice has become increasingly relevant.

The PubMed/MEDLINE and Cochrane Library databases were searched from database inception through March 2026 using the following search strategy:

("tirzepatide" OR "Mounjaro" OR "GLP-1 receptor agonist" OR "incretin therapy") AND ("breast cancer" OR "breast imaging" OR mammography OR ultrasound OR MRI OR "breast density" OR obesity).

Additional manual searches of reference lists from relevant articles were undertaken to identify further studies. Titles and abstracts were screened for relevance, followed by full-text review where appropriate.

Studies were selected based on their relevance to the following areas: pharmacology and metabolic effects of tirzepatide; effects of weight loss on breast tissue composition and palpability; breast cancer incidence, progression, and oncologic outcomes; implications for breast imaging modalities, including mammography, ultrasound, and MRI; and clinical presentation and management of breast symptoms during rapid pharmacologic weight loss.

**Figure 1:**
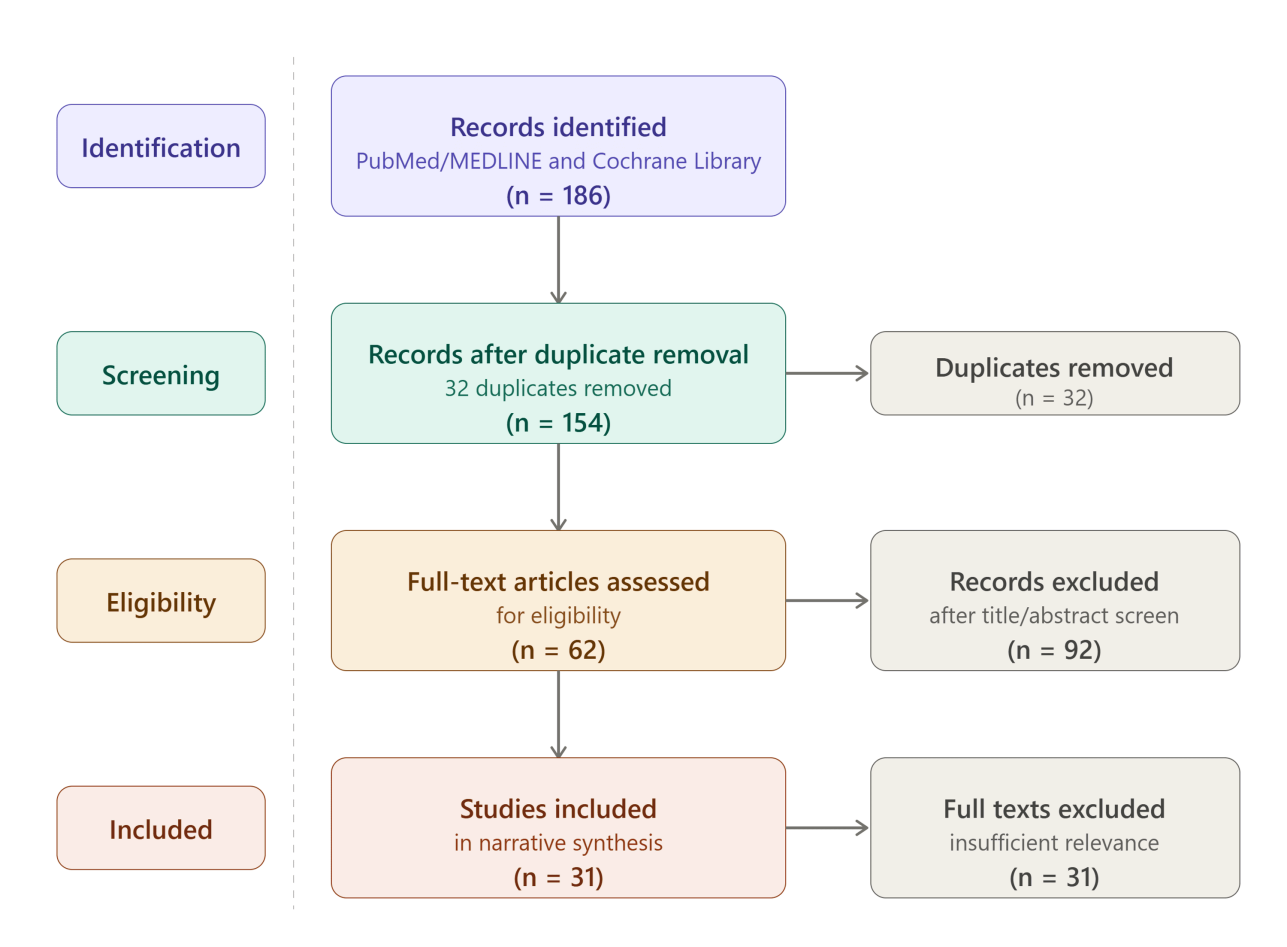
PRISMA-style study selection flow diagram. Databases searched: PubMed/MEDLINE and Cochrane Library, from inception to March 2026. PRISMA: Preferred Reporting Items for Systematic Reviews and Meta-Analyses.

The literature search initially identified 186 records. After the removal of 32 duplicate records, 154 studies remained for title and abstract screening. Following this screening process, 62 articles were selected for full-text assessment. Of these, 31 studies met the predefined inclusion criteria and were ultimately included in the review (Figure 1).

Included references comprised randomised controlled trials, systematic reviews, meta-analyses, observational studies, radiologic investigations, and relevant preclinical research examining the metabolic, oncologic, and imaging-related effects of tirzepatide and GLP-1 receptor agonists. Conference abstracts, translational studies, and early-phase clinical trials were also included where relevant because of the emerging nature of the field. Non-English-language publications and studies lacking direct clinical or scientific relevance to breast disease were excluded.

Evidence was classified according to methodological hierarchy. Level 1 evidence included randomised controlled trials, systematic reviews, and meta-analyses. Level 2 evidence included prospective and retrospective observational studies, registry analyses, and cohort studies. Level 3 evidence comprised preclinical studies, mechanistic investigations, translational research, conference abstracts, and early-phase clinical trials. Clinical conclusions were weighted according to both the level of evidence and the consistency of findings across studies.

A narrative synthesis approach was adopted rather than a formal meta-analysis because of heterogeneity in study design, patient populations, and outcome measures, as well as the limited availability of prospective breast-specific datasets.

Tirzepatide pharmacology and systemic metabolic effects

Tirzepatide is a once-weekly subcutaneous peptide that activates both glucose-dependent insulinotropic polypeptide (GIP) and GLP-1 receptors, enhancing glucose-dependent insulin secretion, suppressing glucagon secretion, delaying gastric emptying, and reducing appetite [1-3]. Across large phase 3 clinical programmes in type 2 diabetes and obesity, tirzepatide has been shown to produce mean weight reductions of approximately 15-22% at higher doses, with substantial reductions in both visceral and subcutaneous adipose tissue and relative preservation of lean mass [4-6].

Body composition imaging from these trials demonstrates that tirzepatide preferentially reduces fat mass relative to lean mass and decreases ectopic fat depots implicated in cardiometabolic risk [5,6]. Because the breast contains both adipose and fibroglandular tissue, these metabolic changes would be expected to reduce breast volume and subcutaneous fat thickness while producing comparatively smaller short- to medium-term changes in fibroglandular tissue volume. Clinically, these changes may resemble those observed following bariatric surgery, although they may occur over a shorter timeframe [14-17].

Oestrogen, menopause, obesity, and incretin-based therapy

Postmenopausal breast cancer risk increases with adiposity through complex interactions involving adipose tissue, sex steroids, insulin, IGF-1, and inflammatory signalling pathways [18]. In obesity, increased aromatase activity within adipocytes enhances oestrogen production, particularly estrone, while chronic low-grade inflammation driven by NF-κB activation and cytokine release may impair effective immunosurveillance [19,20]. Although premenopausal 17β-oestradiol can exert context-dependent anti-inflammatory effects, postmenopausal adipose-derived oestrogen may contribute to a pro-inflammatory and pro-tumourigenic environment [18,20].

GLP-1 receptor agonists and tirzepatide improve insulin sensitivity, reduce hyperinsulinaemia, and downregulate leptin, IGF-1, and other obesity-associated growth signals [4,21].

Collectively, these findings suggest that tirzepatide may modulate metabolic and inflammatory pathways associated with obesity-related breast carcinogenesis; however, direct evidence demonstrating reduced breast cancer incidence or recurrence in humans is currently lacking [21].

Breast tissue composition and weight loss

Breast tissue comprises variable proportions of fibroglandular tissue embedded within adipose stroma. Higher BMI is associated with larger breast volume and lower percentage mammographic density, although absolute dense breast area and breast cancer risk remain increased [13,14]. Excess adipose tissue may reduce the clinical palpability of small or deep lesions, complicate mammographic positioning and compression, and impair image quality [15,16].

Rapid or substantial weight loss reduces breast volume and subcutaneous fat thickness, often without major short-term changes in absolute glandular tissue volume [9,10]. Observational studies following bariatric surgery have demonstrated that benign fibroadenomas, cysts, and nodular glandular tissue may become newly palpable as adipose tissue recedes [14-17]. Patients may also become more aware of asymmetry, costal margins, or prominent ribs, frequently presenting with “new lumps” that subsequently prove benign following triple assessment [17,18]. In addition, the mammographic density category may increase because fibroglandular tissue occupies a greater proportion of a smaller breast volume [9,10].

Given that tirzepatide can produce substantial weight loss over a relatively short timeframe [4,6], breast clinicians may encounter similar patterns of increased benign nodularity, altered contour, and evolving breast composition in women receiving this therapy.

Breast cancer risk and oncologic outcomes

Meta-analyses of randomised controlled trials involving tirzepatide have not demonstrated increased overall cancer risk or a significant increase in breast cancer incidence compared with pooled controls [7,8]. Pharmacovigilance reports and regulatory reviews have likewise not identified a breast cancer safety signal associated with tirzepatide to date [7]. Earlier meta-analyses evaluating GLP-1 receptor agonists in diabetes populations similarly found no consistent increase in breast cancer incidence [11,12].

Retrospective cohort studies of breast cancer patients treated with GLP-1 receptor agonists for diabetes or weight management have reported clinically meaningful weight loss over 6-12 months without currently identified short-term oncologic safety signals regarding recurrence [20,21]. Survivorship cohorts further suggest that GLP-1-induced weight loss may improve cardiometabolic profiles and quality of life [11,22], supporting interest in prospective clinical evaluation within breast oncology populations.

Several ongoing trials are now examining tirzepatide specifically in patients with early-stage hormone receptor-positive breast cancer [23,24]. Studies involving patients with minimal residual disease detected through circulating tumour DNA are exploring whether tirzepatide-mediated weight loss may influence metabolic risk factors, ctDNA dynamics, and recurrence-related outcomes when combined with standard endocrine therapy [24]. Other trials are evaluating tirzepatide safety, tolerability, and weight loss efficacy in patients with hormone receptor-positive, HER2-negative early breast cancer receiving adjuvant treatment [23]. These studies may help define the role of tirzepatide as a metabolic adjunct in breast oncology rather than as a direct anticancer therapy.

Implications for breast imaging

Mammography

Weight loss associated with tirzepatide may produce several anticipated mammographic changes.

A relative increase in qualitative breast density may occur as fibroglandular tissue occupies a greater proportion of overall breast volume, potentially resulting in upward shifts in Breast Imaging Reporting and Data System (BI-RADS) density category even when absolute glandular volume remains stable [9,13].

Reduction in subcutaneous and retromammary adipose tissue may render Cooper’s ligaments, ribs, and pectoralis musculature more conspicuous, potentially contributing to palpable but benign findings [10,14].

Reduced compressed breast thickness may theoretically improve image sharpness and decrease scatter, potentially improving visualisation of subtle architectural distortion and microcalcifications, particularly in previously obese women [15,16].

Emerging studies using AI-based quantitative density assessment and radiomic analysis following substantial GLP-1-mediated weight loss suggest that although qualitative breast density may increase, quantitative radiomic features previously associated with breast cancer risk may evolve differently, potentially reflecting broader metabolic and parenchymal changes [9,25]. These findings highlight the importance of interpreting density changes within the broader context of pharmacologically induced weight loss rather than as isolated surrogate markers of breast cancer risk.

Ultrasound

Tirzepatide-induced weight loss may alter sonographic breast appearance as adipose lobules diminish, producing relatively more echogenic breast parenchyma and bringing glandular tissue closer to the skin surface and chest wall [10,15]. Small hypoechoic lesions may therefore become more conspicuous in thinner breasts, although benign heterogeneity of fibroglandular tissue may also appear more prominent.

In women with previous surgery or radiotherapy, reduction in adipose tissue may accentuate scar tissue, fat necrosis, or fibrosis, which can appear more conspicuous both clinically and sonographically [9,16].

Sonographers may need to adjust imaging depth and focal settings to accommodate reduced tissue thickness and should remain aware that new nodularity following major weight loss may frequently correspond to benign glandular tissue.

MRI and Functional Imaging

There is currently no evidence that tirzepatide alters gadolinium pharmacokinetics or directly affects enhancement characteristics on breast MRI [9]. However, background parenchymal enhancement and diffusion-related imaging parameters may be influenced by hormonal status, adiposity, and changes in breast composition [9,15].

Ongoing tirzepatide studies involving high-risk women and patients with early breast cancer are incorporating MRI and tissue biomarkers to explore whether pharmacologic weight loss influences background enhancement patterns and radiomic signatures potentially relevant to breast cancer risk assessment [24,25].

At present, there is insufficient evidence to support modification of standard MRI acquisition protocols or interpretation criteria; however, documenting recent major weight loss and concurrent endocrine or metabolic therapies may assist interpretation of interval imaging changes. Importantly, no completed prospective breast imaging studies specifically evaluating tirzepatide-associated breast compositional change are currently available.

Clinical implications in breast surgery

Triple Assessment and Symptomatic Presentations

Women receiving tirzepatide may present with newly palpable breast findings representing pre-existing benign lesions previously rendered impalpable by adipose tissue, prominent costal margins or ribs, or nodular fibroglandular tissue that becomes more distinct within a smaller breast volume [14,17]. Despite this evolving presentation pattern, standard triple assessment, including clinical examination, appropriate imaging, and biopsy where indicated, remains essential. There are currently no recommendations to modify screening intervals, delay imaging, or alter biopsy thresholds solely because of tirzepatide or other GLP-1/GIP receptor agonist use [7,20].

Clinicians should nevertheless enquire specifically about recent weight change and incretin-based therapies and explain to patients that benign nodularity and anatomical prominence may become more apparent during substantial weight loss.

This balanced approach helps maintain diagnostic vigilance while minimising unnecessary anxiety, imaging escalation, and intervention within a rapidly expanding population receiving metabolic therapies.

Oncologic Practice Points

For oncologists and multidisciplinary teams, tirzepatide represents an emerging option for weight management in selected women with breast cancer or elevated metabolic risk, particularly those with obesity and metabolic syndrome [18,21,22]. However, its use should be integrated with careful consideration of sarcopenia, bone health, nutritional status, and cardiovascular risk.

Current trial and observational data do not demonstrate increased breast cancer incidence or recurrence associated with tirzepatide or GLP-1 receptor agonists [7,8,12-15,25], although long-term follow-up remains limited. Shared decision-making and continued oncologic surveillance therefore remain important.

Emerging biomarkers, including circulating tumour DNA, advanced imaging metrics, and tissue biomarkers being evaluated in ongoing tirzepatide trials, may further clarify whether metabolic interventions influence recurrence-related outcomes [24].

Radiology Reporting and Workflow

Breast imaging services may adapt workflow practices by documenting recent major weight change and GLP-1/GIP therapy use on imaging request forms and by recognising expected alterations in breast density, parenchymal pattern, and anatomical prominence during interpretation of interval studies [13-16].

Appropriate patient counselling is also important, as new palpable findings during rapid weight loss frequently prove benign following assessment [14,17], while all new findings should nevertheless continue to undergo standard diagnostic evaluation.

Implications for surgical decision-making and anaesthesia

The increasing use of tirzepatide and other incretin-based therapies for the management of obesity and type 2 diabetes has important implications for perioperative planning in breast surgery, particularly in patients undergoing oncoplastic resection or reconstructive procedures.

Surgical Decision-Making and Oncoplastic Planning

Tirzepatide-induced weight loss is frequently substantial and may occur over a relatively short period, resulting in dynamic changes in breast volume, contour, tissue composition, and symmetry [4-6]. These evolving anatomical changes may influence operative planning and the selection of appropriate oncoplastic techniques aimed at optimising both oncologic and aesthetic outcomes.

In breast-conserving surgery, reductions in breast volume may alter tumour-to-breast size ratios, potentially improving the feasibility of breast conservation in some patients while complicating volume-displacement strategies in others [13,14]. Established oncoplastic principles emphasise tailoring surgical technique according to breast size, tumour location, and degree of ptosis, all of which may evolve during rapid pharmacologic weight loss [26]. For example, relatively small tumours in previously large or ptotic breasts may still require advanced oncoplastic approaches, including therapeutic reduction mammoplasty, to preserve breast contour and facilitate adjuvant radiotherapy.

However, continued postoperative weight loss may subsequently result in further volume reduction, asymmetry, recurrent ptosis, or contour deformity, potentially affecting long-term aesthetic outcomes. Where oncologically feasible, relative weight stabilisation may therefore be considered during operative planning. In selected patients, bilateral symmetrisation procedures at the index operation may also help reduce anticipated postoperative asymmetry.

In reconstructive surgery, particularly implant-based reconstruction, reductions in subcutaneous tissue thickness may reduce soft-tissue implant coverage and increase implant visibility, contour irregularity, and rippling. Similarly, outcomes following autologous reconstruction and fat grafting may be influenced by ongoing metabolic changes, fat redistribution, and variability in graft retention [27]. These considerations highlight the importance of comprehensive preoperative counselling regarding the potential impact of continued pharmacologic weight loss on reconstructive outcomes and long-term breast morphology.

Although existing literature confirms that breast size, body composition, and tumour-to-breast ratio are important determinants of oncoplastic surgical planning [26,28], no studies have specifically evaluated the effects of rapid pharmacologic weight loss on these parameters. Current considerations are therefore based largely on extrapolation from established oncoplastic principles and clinical experience rather than prospective surgical outcome data, highlighting an emerging area of importance in contemporary breast surgery practice.

Anaesthetic Considerations

GLP-1 receptor agonists, including tirzepatide, are associated with delayed gastric emptying, which has raised concerns regarding an increased risk of perioperative aspiration [2,3]. Recent guidance from anaesthetic societies recommends consideration of withholding these agents prior to elective surgery, although recommendations vary depending on dosing schedules and individual patient risk factors [29,30].

For patients receiving weekly formulations such as tirzepatide, temporary discontinuation prior to surgery may reduce the risk of residual gastric contents at the time of induction. Preoperative assessment should include specific enquiry regarding GLP-1/GIP agonist use, and anaesthetic management may need to be adapted accordingly. In higher-risk patients, strategies such as rapid sequence induction or additional airway precautions may be appropriate [29,30].

Multidisciplinary Considerations

These evolving factors highlight the importance of multidisciplinary coordination between breast surgeons, anaesthetists, and perioperative teams. Incorporating medication history, recent weight change, and treatment trajectory into preoperative planning can help optimise both oncologic safety and aesthetic outcomes while minimising perioperative risk.

Future research directions

Future research should focus on prospective evaluation of breast imaging and oncologic outcomes in women receiving tirzepatide and other incretin-based therapies. Key priorities include longitudinal cohort studies examining changes in mammographic density, background parenchymal enhancement, and radiomic breast signatures before and after pharmacologic weight loss, correlated with metabolic markers and clinical outcomes [13,25,31].

Large registry-linkage studies are also needed to evaluate long-term breast cancer incidence, stage at diagnosis, recurrence patterns, and survival outcomes among women exposed to tirzepatide and GLP-1 receptor agonists [7,8,12,24]. In parallel, ongoing and future clinical trials in breast cancer survivors and high-risk populations should assess whether tirzepatide-mediated weight loss favourably influences cardiometabolic health, circulating tumour DNA clearance, recurrence risk, and long-term survival [24].

Further research is also required to better define the impact of rapid pharmacologic weight loss on surgical planning, reconstructive outcomes, and radiological interpretation. Qualitative studies exploring patient and clinician understanding of new breast symptoms and imaging changes during GLP-1/GIP therapy may additionally help optimise patient counselling and reduce unnecessary anxiety and investigation.

Limitations of the review

As a narrative review, study selection was not performed according to a predefined systematic review protocol, introducing the possibility of selection bias. Although a structured literature search was undertaken, relevant studies may have been inadvertently omitted. Consequently, the conclusions presented should be interpreted as a synthesis of current evidence rather than a definitive quantitative assessment.

Evidence specifically evaluating radiological performance metrics, including sensitivity, specificity, recall rates, and interval cancer detection, in patients undergoing rapid pharmacologic weight loss remains sparse. Current radiological interpretations are therefore based largely on indirect evidence, extrapolation from bariatric surgery literature, and emerging radiomic analyses rather than dedicated prospective breast imaging studies.

Long-term human data evaluating the relationship between tirzepatide and breast cancer incidence, recurrence, and imaging outcomes remain limited. Most available trials were designed primarily around metabolic endpoints and therefore provide limited follow-up regarding breast cancer incidence, recurrence, survival, or imaging changes. Consequently, the absence of an identified breast cancer safety signal should not be interpreted as definitive evidence of long-term safety, and continued surveillance through prospective studies and registry-based analyses remains necessary.

Finally, several ongoing clinical trials investigating tirzepatide in breast cancer populations remain immature, and evidence regarding its effects on recurrence, survival, and biomarkers such as circulating tumour DNA is still preliminary. Furthermore, the major tirzepatide randomised trials were not designed with breast-specific endpoints, and female-only subgroup data relevant to breast oncology have not been systematically reported.

Despite these limitations, this review highlights an important and rapidly evolving intersection between metabolic therapy, breast imaging, and breast oncology, while identifying several priorities for future clinical and translational research.

## Conclusions

Tirzepatide-associated weight loss alters breast tissue composition in ways that may increase the palpability of pre-existing benign lesions and modify mammographic density and ultrasound appearances, without current evidence suggesting impaired diagnostic accuracy. Available randomised-trial, observational, and pharmacovigilance data do not demonstrate an increased risk of breast cancer with tirzepatide or other GLP-1 receptor agonists, while preclinical studies suggest potential attenuation of obesity-associated mammary tumour progression through metabolic and inflammatory modulation.

At present, breast clinics should continue standard triple assessment pathways while incorporating recent weight change and incretin-based therapy use into clinical history-taking, imaging interpretation, and perioperative planning. Clinicians should remain vigilant for malignancy while recognising that rapid pharmacologic weight loss may increase the clinical prominence of previously occult benign breast findings.

High-quality prospective imaging, surgical, and oncologic studies are now required to define the long-term implications of tirzepatide use for breast cancer detection, breast composition, reconstructive outcomes, recurrence risk, and survivorship care in an increasingly prevalent patient population.
