# Correction: Clinical Implications of Mounjaro (Tirzepatide) for Breast Cancer Detection and Management: A Narrative Review

**DOI:** 10.7759/cureus.c457

**Published:** 2026-06-30

**Authors:** Raafat Mady, Habib Tafazal, Haitham Soliman, Ayman Ramadan, Mohamed Hajaj

**Affiliations:** 1 Department of Breast Surgery, Kettering General Hospital, Kettering, GBR; 2 Department of Breast Surgery, Blackrock Clinic, Dublin, IRL; 3 Department of Medical Oncology, Kettering General Hospital, Kettering, GBR; 4 Radiology Department, Breast Cancer Center, American Hospital, Dubai, ARE

This article has been corrected to change Dr. Mohamed Hajaj’s affiliation to: Radiology Department, Breast Cancer Center, American Hospital, Dubai, UAE.

